# Connectivity of the Habitat-Forming Kelp, *Ecklonia radiata* within and among Estuaries and Open Coast

**DOI:** 10.1371/journal.pone.0064667

**Published:** 2013-05-23

**Authors:** Melinda A. Coleman

**Affiliations:** 1 Centre for Marine BioInnovation, University of New South Wales, Sydney, New South Wales, Australia; 2 New South Wales Fisheries, Department of Primary Industries, Coffs Harbour, New South Wales, Australia; 3 National Marine Science Centre, Southern Cross University, Coffs Harbour, New South Wales, Australia; The Australian National University, Australia

## Abstract

With marine protected areas being established worldwide there is a pressing need to understand how the physical setting in which these areas are placed influences patterns of dispersal and connectivity of important marine organisms. This is particularly critical for dynamic and complex nearshore marine environments where patterns of genetic structure of organisms are often chaotic and uncoupled from broad scale physical processes. This study determines the influence of habitat heterogeneity (presence of estuaries) on patterns of genetic structure and connectivity of the common kelp, *Ecklonia radiata*. There was no genetic differentiation of kelp between estuaries and the open coast and the presence of estuaries did not increase genetic differentiation among open coast populations. Similarly, there were no differences in level of inbreeding or genetic diversity between estuarine and open coast populations. The presence of large estuaries along rocky coastlines does not appear to influence genetic structure of this kelp and factors other than physical heterogeneity of habitat are likely more important determinants of regional connectivity. Marine reserves are currently lacking in this bioregion and may be designated in the future. Knowledge of the factors that influence important habitat forming organisms such as kelp contribute to informed and effective marine protected area design and conservation initiatives to maintain resilience of important marine habitats.

## Introduction

Knowledge of the physical and biological factors that structure patterns of connectivity and dispersal of nearshore marine organisms is critical for effective marine conservation [1], [2]. With marine protected areas (MPAs) being established worldwide to protect marine biodiversity and ecological processes, there is a pressing need to understand the interplay between the physical setting in which these areas are placed, relative to biological factors that determine patterns of dispersal of important marine organisms. This will ensure that MPAs are optimally designed to maintain connectivity among spatially separated populations and allow informed, adaptive management.

Marine Protected Areas (MPAs) are commonly designated in nearshore marine environments which are inherently physically dynamic and spatially complex. Further, individual MPAs are designed on local spatial scales where dispersal and connectivity of marine organisms is rarely structured by the broad scale physical processes traditionally known to impart structure along entire coastlines [e.g. 3]. Patterns of genetic structure of organisms inhabiting nearshore marine environments and on such local spatial scales often appears chaotic, bearing weak relationships to distances among populations or overarching oceanographic conditions. Instead, factors such as location-specific habitat discontinuities or availability [e.g. 4], [5], hydrodynamics [e.g. 6], [7] and coastal topography [e.g. 8] may structure patterns of genetic connectivity on the regional scales at which MPAs are typically designed.

Nearshore marine environments are often characterised by rocky reefs that are interspersed with habitat discontinuities and potential barriers to dispersal such as sandy beaches, estuaries and bays. Such heterogeneities of habitat have the potential to restrict dispersal and gene flow of rocky reef organisms and create complex patterns of genetic structure among populations. The presence of estuaries and beaches has been shown to increase genetic differentiation among rocky coast populations of intertidal [9] and subtidal algae [3], invertebrates [10], and fish [11], [12], [13], [14]. Estuaries have also been suggested to restrict gene flow between populations living inside versus outside these bodies of water [10], [14]. Water exchange between estuaries and the open coast is often restricted and characterised by plumes of water that oscillate back and forth with the tides. This may restrict the dispersal of propagules between estuarine and open coast habitats. Conversely, there may be much interannual variability in the flushing rates of estuaries and/or physical conditions that influence phenology (e.g. reproductive timing) and subsequent transport of propagules between open coast and estuarine sites [15].

This study assessed the extent to which the presence of major estuaries restricts genetic connectivity of the cosmopolitan, habitat-forming kelp, *Ecklonia radiata*. *Ecklonia radiata* (C. Agardh) J. Agardh is the dominant form of biogenic habitat on Australia's temperate reefs [16] and is a true “foundation species” [17] because its presence largely determines associated community structure [18], [19], [20]. *E. radiata* mostly inhabits open coast sites and is the most abundant macroalga on temperate rocky reefs of temperate Australia. However, in estuarine habitats this species can live on the small amount of available hard substrata and in the upper reaches of estuaries where suitable rocky substrata is often lacking, it is commonly found growing on artificial structures such as pontoons, pilings and breakwalls. *E. radiata* has a typical Laminarian alternation of generations life history strategy with conspicuous, macroscopic sporophytes (spore producing individuals) alternating with microscopic gametophytes (gamete producing individuals [21]). Because microscopic gametophytes have never been found in the field, this study necessarily characterises patterns of genetic structure of sporophytes. Patterns of genetic structure are therefore the combined effects of dispersal of zoospores and sperm. Depending on the dispersal distances and settlement of zoospores, kelp can, therefore, both outcross and potentially self fertilise [22]. Fertile sporophytes may also disperse when they are removed from the substratum during storms [23].

East coast populations of this species exhibit low “chaotic”, genetic differentiation across approximately 800 km of coastline with no relationship to distances among sites or predictions of broad scale oceanographic dispersal [24]. This might be partly explained by the major estuarine systems along some portions of this coast, which have the potential to create complex patterns of dispersal among open coast populations of this kelp. Moreover, this species inhabits both open coast and, to a lesser extent, estuarine hard substrata and hydrodynamics or phenological factors may potentially act as a barrier to dispersal between these habitats. Hydrodynamic conditions within estuaries vary greatly depending on the position and distance of sites from the opening and this can also influence patterns of genetic structure [25]. Therefore, this study also examined whether the smaller *E. radiata* populations in estuaries would be genetically differentiated from nearby open coast sites and whether the proximity to estuarine entrance would influence genetic structure. Understanding how the physical coastal environment influences patterns of dispersal and connectivity in marine organisms, particularly ecologically important habitat forming species such as kelp, will be an important component for designing future MPAs for this region and rezoning existing MPAs elsewhere along the coast.

## Materials and Methods

**Figure 1:**
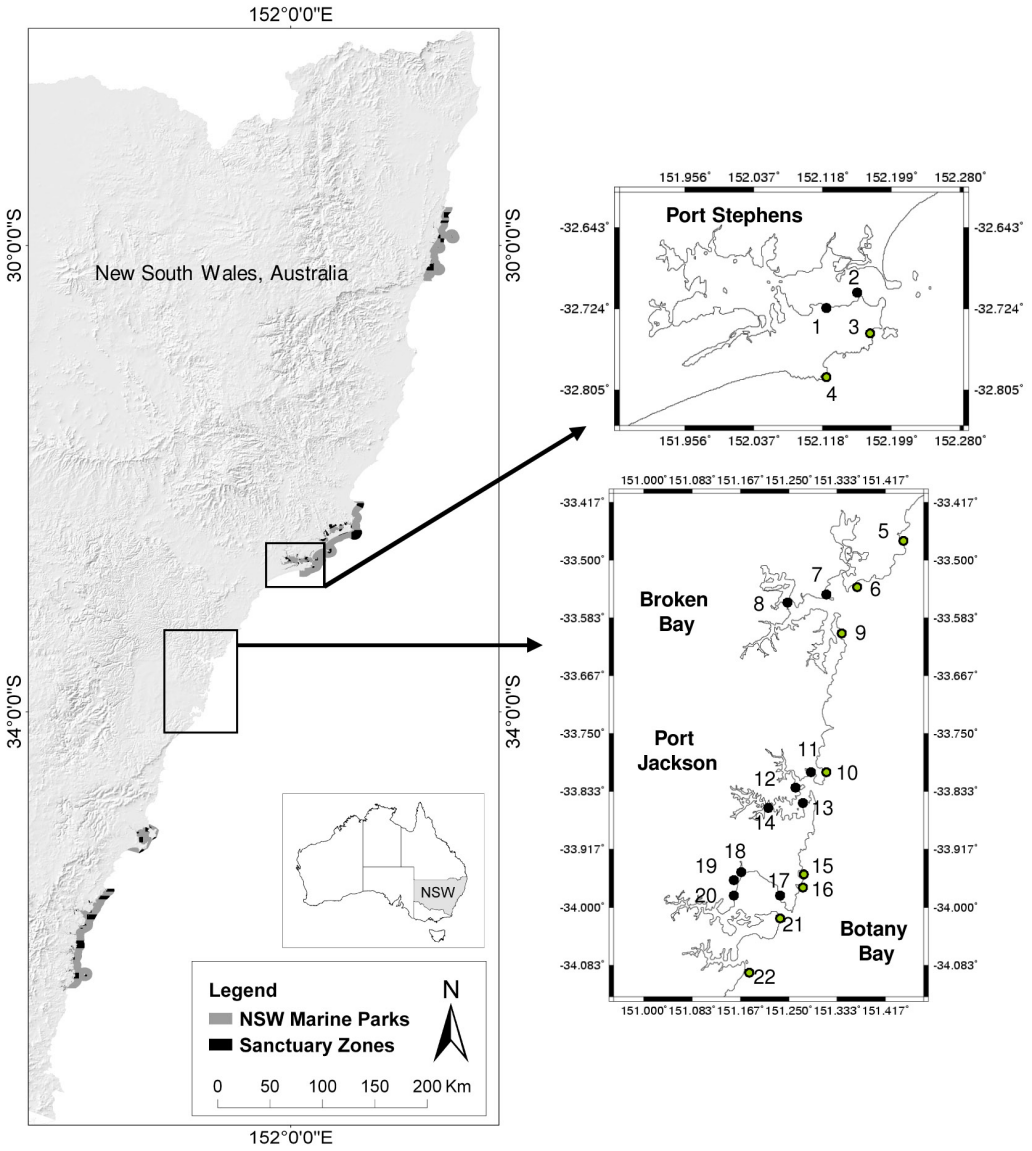
Map of southeastern Australia showing the estuaries and open coast sites sampled. Black symbols represent the position of sites within bays/estuaries and green symbols represent open coast sites. Site numbers correspond to Table 2. Existing networks of marine reserves ( = sanctuary zones) within each of 4 marine parks are shown.

*E. radiata* sporophytes were sampled from small sections of rocky substrata within 4 estuaries and from large kelp forests on nearby open reef sites along approximately 200 km of the coast of south-eastern Australia in June 2006. From north to south these estuaries were Port Stephens (PS: S32.7185, E152.10623), Broken Bay (BB: S33.5486, E151.2294), Port Jackson (PJ: S33.8456, E151.2813) and Botany Bay (BotB: S34.01675, E151.23093) (Fig. 1, Table 1). This section of coastline was chosen because (i) it has 4 of the largest estuaries on the east coast of Australia within a small geographic distance, (ii) marine parks are potentially warranted in this area to complete a comprehensive coverage of bioregions and knowledge of genetic structure of this important habitat forming species would aid in MPA design and (iii) being the most urbanised coastline in Australia *E. radiata* may be at risk of substantial declines [26], [27]. These estuaries differ in historical patterns of formation and water movement with Port Stephens and Broken Bay being wave-dominated estuaries and Port Jackson and Botany Bay being tide-dominated estuaries (Table 1). Two to 4 sites (separated by 1–11 kilometres) were sampled within each estuary and these were often on artificial hard substrata. *E. radiata* was also sampled from large areas of kelp forest at open coast sites that were similar distances apart (6–24 km) and positioned to the north and south of the entrance to each estuary (Fig. 1). All estuarine and open coast sites were within 1 to 5 m depth. Portions of the unfouled, lower laterals of between 18 and 32 mature (stage 3, [28]) *E. radiata* individuals were randomly collected and returned to the laboratory on ice. Material was rinsed in freshwater, blotted dry on paper towels and dried in silica gel. DNA was extracted and 6 microsatellite markers [29] were amplified and genotyped as in [30]. All necessary permits were obtained for the described study which complied with all relevant regulations. Permits were issued by The NSW Department of Primary Industries, Fisheries and all work was conducted in state waters.

**Table 1:** Characteristics of the 4 estuaries sampled.

	Area Rocky Reef (km^2^)	Total Area (km^2^)	Mean Tidal Flow (10^6^ m^3^)	Estuary Classification
**Port Stephens**	1.36	128.36	157.50	Wave
**Broken Bay**	0.01	47.47	195.00	Wave
**Port Jackson**	0.52	50.47	82.33	Tide
**Botany Bay**	0.49	38.31	No data	Tide

Mean flow rate is the mean volume of water that flows into and out of the estuary during flood and ebb tides, measured close to the oceanic entrance. Estuary classification indicates the dominant force shaping the hydrodynamics of the estuary. Data are from the Ozcoasts database (http://www.ozcoasts.org.au, accessed 2012).

Prior to conducting statistical analyses, data were checked for typographical and genotyping errors using Microchecker [31]. Patterns of genetic diversity were characterized using a number of different descriptive measures. The total number of alleles, number of unique alleles, allele frequencies, and observed (*H*_o_) and expected heterozygosities (*H*_e_) were generated using GENETIX ver. 4.04 [32] for each population. In addition, data were tested for linkage disequilibrium and Hardy-Weinberg equilibrium at each locus and across all loci using FSTAT 1.2 [33]. Genetic structure was estimated by testing Weir and Cockerham's *F*_ST_ and *F*_IS_ estimates [34] using permutation tests (1000 permutations, FSTAT 1.2, 33). Pairwise *F*_ST_ estimates were also estimated between sites. A sequential Bonferroni correction [35] was used when examining significance levels for pairwise tests. Random mating was not assumed in these analyses so genotypes (rather than alleles) were permuted. To determine the percentage of variation explained at each spatial scale, analyses of molecular variance (AMOVA) were done in ARLEQUIN ver. 3.00 [36] using *F* statistics. A stepwise mutation model was not assumed and *P*<0.05 was used. We identified potential first generation migrants using GeneClass 2 [37] as an indirect measure of past dispersal. Assignment tests are limited by the number of potential “source” populations sampled. That is, based on genotype frequencies, an individual will be assigned to one (the most likely) source population, even if there is a low probability of it actually coming from that population. To assess this problem, Gene Class 2 uses Monte Carlo resampling techniques to compute the probability of an individual belonging to each given source population. Tests were done using the Rannala and Mountain (1997) Bayesian method of computing genotypes [38] because this method often performs better than distance based methods [39], [40], particularly when the number of loci and number of replicates are small [39]. Individuals were considered migrants if they had a probability of coming from a population other than the one in which they were sampled of *P*<0.01. Tests of isolation by distance for each estuary and surrounding open coast sites were done via Mantel tests using the program IBD [41]. Geographic distances between each pair of sites were calculated using shoreline distances. Linear regression lines were fitted to figures for graphical representation.

## Results

There was no evidence of null alleles or linkage disequilibrium at any locus and the majority of populations and loci were in Hardy-Weinberg equilibrium except a few sites at a few loci but there were no consistent patterns. The total number of alleles was similar between estuarine and open coast sites (t-test *P*>0.05, Table 2). *F*_IS_ estimates were mostly non-significant indicating random mating and there were no apparent patterns in the magnitude of *F*_IS_ estimates between open coast and estuarine sites (Table 2). Although significant *F*_IS_ estimates were mostly positive indicative of inbreeding, both open coast sites at Port Stephens, however, had significantly negative *F*_IS_ estimates indicating excess of heterozygtes.

**Table 2:** Number of individuals sampled (*n*), total number alleles (*n*_a_), expected and observed heterozygosity and *F*_IS_ (a measure of inbreeding within populations) for each location.

Estuary	Position		Site	*n*	*n*_a_	H_e_	H_o_	*F*_IS_	
Port Stephens	**Inside**	**1**	**Marina 1**	32	19	0.308	0.344	−0.102	
	*F*_ST_ = 0.001	**2**	**Marina 2**	32	19	0.330	0.314	0.063	
	**Outside**	**3**	**Boat Harbour**	32	18	0.349	0.469	−0.330	*
	*F*_ST_ = 0.002	**4**	**Anna Bay**	32	17	0.323	0.443	−0.355	*
Broken Bay	**Inside**	**7**	**Pearl Beach**	30	18	0.357	0.328	0.099	
	*F*_ST_ = 0.09	**8**	**Brooklyn**	32	15	0.388	0.365	0.076	
	**Outside**	**9**	**Palm Beach**	22	18	0.374	0.366	0.042	
	*F*_ST_ = 0.037	**5**	**Terrigal**	32	18	0.320	0.333	−0.027	
		**6**	**Putty Beach**	32	19	0.405	0.401	0.027	
Port Jackson	**Inside**	**13**	**Vaucluse**	31	19	0.418	0.312	0.270	*
	*F*_ST_ = 0.022	**14**	**Balmain**	18	15	0.331	0.321	0.051	
		**12**	**Chowder Bay**	32	18	0.385	0.344	0.123	
		**11**	**Fairlight**	32	19	0.360	0.266	0.264	*
	**Outside**	**10**	**Manly**	32	18	0.413	0.376	0.097	
	*F*_ST_ = 0.038	**15**	**Coogee**	31	19	0.368	0.290	0.227	*
Botany Bay	**Inside**	**17**	**Bare Island**	32	14	0.340	0.250	0.280	*
	*F*_ST_ = 0.025	**18**	**Cooks River opening**	32	17	0.349	0.365	−0.028	
		**19**	**Groyne 1**	30	16	0.313	0.356	−0.120	
		**20**	**Groyne 2**	32	16	0.356	0.390	−0.091	
	**Outside**	**16**	**Malabar**	32	16	0.371	0.255	0.327	*
	*F*_ST_ = 0.013	**21**	**Sutherland**	32	20	0.386	0.333	0.152	
		**22**	**Cronulla**	32	21	0.339	0.318	0.078	

*F*_ST_ estimates for inside and outside each estuary are shown. Numbers preceding site names correspond to Figure 1 map.

* = P<0.0005.

*F*_ST_ estimates were similar among estuarine (mean *F*_ST_ = 0.035) and among open coast sites (mean *F*_ST_ = 0.023, t-test *P*>0.05). Similarly, comparisons of estimates of *F*_ST_ for each individual estuary were similar to nearby open coast sites (Table 2). There was no clear pattern in the magnitude of genetic differentiation between estuaries and open coast sites, with differentiation among estuarine sites sometimes greater than (Botany Bay, Broken Bay) and sometimes smaller than (Port Jackson, Port Stephens) nearby open coast sites (Table 2).

AMOVA for each individual estuary revealed that estuarine and nearby open coast sites were only genetically different at Port Stephens (Table 3). This was most likely driven by 1 open coast site (Boat Harbour) which was different from all estuarine sites (pairwise tests, Table 4). Most variation was explained among individuals within sites with a small amount of variation explained among sites (Table 3). Pairwise tests confirmed this result with few significant differences between sites (Table 4).

**Table 3:** Analysis of Molecular Variance (AMOVA) averaged over all loci, between estuaries and open coast sites for each estuary.

	Source of variation	d.f.	SS	Variance component	% of variation	
**(a)**	**Port Stephens**					
	Between Estuary and Open coast	1	7.18	0.0504	4.82	*
	Among sites within	2	1.47	0.0042	0.40	
	Among individuals within sites	252	251.24	0.9976	95.57	*
**(b)**	**Broken Bay**					
	Between Estuary and Open coast	1	3.84	0.0101	0.85	
	Among sites within	3	15.37	0.0686	5.79	*
	Among individuals within sites	291	327.16	1.1257	95.06	*
**(c)**	**Port Jackson**					
	Between Estuary and Open coast	1	2.98	0.0023	0.19	
	Among sites within	4	12.78	0.0355	2.96	***
	Among individuals within sites	346	401.95	1.1673	97.24	*
**(d)**	**Botany Bay**					
	Between Estuary and Open coast	1	2.20	0.0014	0.13	
	Among sites within	5	12.57	0.0229	2.09	*
	Among individuals within sites	437	466.74	1.0696	98.04	*

* = P<0.00001.

**Table 4:** Pairwise *F*_ST_ estimates between all pairs of sites within estuaries and nearby open coast sites, significant values after the Bonferroni sequential correction are in bold.

PS				
	*Marina1*	*Marina2*	Boat H	Anna
*Marina1*	0			
*Marina2*	0.0009	0		
Boat H	**0.0569**	**0.0565**	0	
Anna	0.0342	0.0393	0.0017	0

BB					
	*Pearl*	*Brooklyn*	Palm Bch	Terrigal	Putty Bch
*Pearl*	0				
*Brooklyn*	**0.0902**	0			
Palm Bch	0.0229	**0.0848**	0		
Terrigal	0.0052	**0.1264**	**0.0812**	0	
Putty Bch	0.0078	**0.0541**	0.0148	0.0219	0

PJ						
	Manly	Coogee	*Vaucluse*	*Chowder*	*Fairlight*	*Balmain*
Manly	0					
Coogee	0.0378	0				
*Vaucluse*	0.0078	**0.0532**	0			
*Chowder*	**0.0456**	0.0031	0.0270	0		
*Fairlight*	0.0381	0.0072	0.0296	0.0042	0	
*Balmain*	0.0191	0.0246	0.0237	0.0405	0.0068	0

BotB							
	Malabar	Cronulla	Suther	*Bare Isld*	*Cook*	*Groyne1*	*Groyne2*
Malabar	0						
Cronulla	**0.0338**	0					
Suther	0.0122	0.0164	0				
*Bare Isld*	0.0100	0.0239	0.0047	0			
*Cook*	0.0070	**0.0328**	0.0164	**0.0443**	0		
*Groyne1*	0.0017	0.0370	0.0070	0.0348	0.0138	0	
*Groyne2*	0.0213	0.0216	0.0216	0.0398	0.0008	0.0110	0

Sites inside estuaries are in italics. Estuary abbreviations are as in materials and methods.

**Figure 2:**
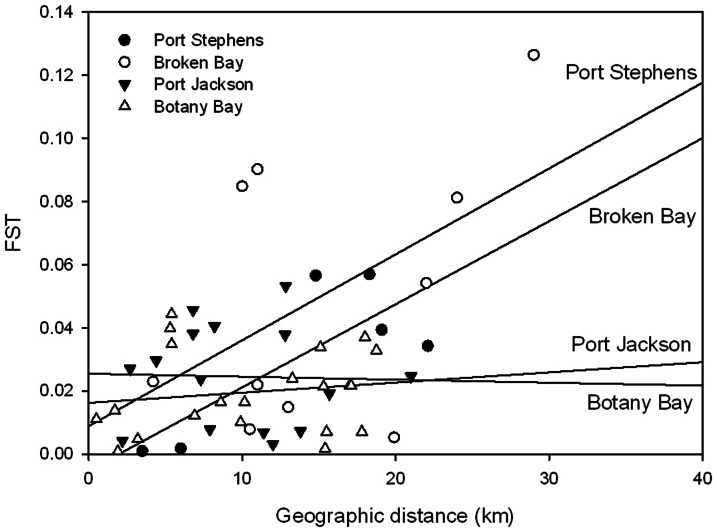
Relationship between genetic differentiation (*F*_ST_) and distances between sites (km). Regression lines are shown for each estuary.

Pairwise tests also showed that sites that were near the entrance of estuaries were no more similar to open coast sites than those that were positioned further inside estuaries (Table 4). However, in 1 estuary (Broken Bay) the site furthest away from the entrance (Brooklyn) was genetically different from all other sites whether inside or outside the estuary (Table 4). Tests for isolation by distance were not significant with no correlation between genetic differentiation and geographic distances for any estuary (Mantel tests PS: *Z* = 3.40 *r* = 0.79, BB: *Z* = 9.36, *r* = 0.50, PJ: *Z* = 3.55 *r* = −0.03, BotB: *Z* = 4.60 *r* = 0.15). This result may be a reflection of low replication as graphical representation of data (Fig. 2) suggested a trend for positive relationships between genetic differentiation and geographic distance for the 2 wave dominated estuaries (Port Stephens and Broken Bay) but not for the 2 tide dominated estuaries (Port Jackson and Botany Bay).

Estimates of mean pairwise *F*_ST_ for several other ∼200 km sections of rocky coastline without major estuaries to the north (Coffs Harbour to Port Macquarie *F*_ST_ = 0.048) and south (Wollongong to Batemans *F*_ST_ = 0.049, data reanalysed from 24) were greater than for the region in the present study (*F*_ST_ = 0.023 for open coast sites).

Tests for first generation migrants revealed that 11% of all individuals are likely migrants from another site (*P*<0.01). These represented 9% of all individuals from estuaries and 13% of individuals from the open coast. Individuals growing in estuaries that were identified as migrants were mostly (62%) assigned to populations of origin that were open coast sites rather than estuarine sites. Conversely, individuals growing on the open coast that were identified as migrants were mostly assigned to other open coastal sites (66%).

## Discussion

To design and manage effective networks of MPAs we need to understand the interplay between the physical settings in which these areas are being designated, relative to the biology and ecology of important organisms. A key consideration in designing networks of marine reserves is to maintain connectivity among populations [1], particularly in a future of increasing anthropogenic impacts. Knowledge of how connectivity is influenced by the physical environment in which specific MPAs are to be placed is thus critical for their success. This study examined how the presence of habitat discontinuities in the form of major estuaries, influences regional connectivity of one of the most common and abundant habitat forming kelps in Australia, *E. radiata* and along a section of coastline where future MPAs are warranted to complete a comprehensive coverage of bioregions.

The presence of estuaries did not restrict gene flow among rocky reef populations of this kelp and estimates of connectivity along rocky coasts interspersed with estuaries were actually smaller than similar sections of coastline where no major estuaries exist. Thus, the prediction that genetic differentiation would be greater along coastlines where suitable rocky reef habitat is interspersed with estuaries was not supported. This suggests that the presence of estuaries are not a factor in determining levels of genetic differentiation along rocky coastlines and do not appear to restrict dispersal and gene flow for this species. Indeed, given that this kelp species can also inhabit estuarine areas, the presence of small estuarine populations may instead increase connectivity among nearby open coast populations via provision of additional habitat (including artificial substrata) and populations that would otherwise not exist. In addition, the unique environmental conditions (hydrodynamics, temperature, nutrients) within estuaries may extend or alter phenological factors (such as timing of reproduction) relative to open coast populations [e.g. 42], effectively resulting in broader temporal gene flow along the surrounding coastline. Indeed, approximately 34% of migrants from open coast sites were assigned to estuarine populations of origin. Incorporating estuarine areas into networks of MPAs is, therefore, an important consideration for the long term persistence and conservation of rocky reef organisms. Estuarine areas are currently an integral part of the design of MPAs (or marine parks) along the coast of NSW and this study supports their importance as critical links to open coastal areas.

Interestingly, there was generally no genetic differentiation between open coast and estuarine populations of this species indicating that small populations of kelp within estuaries are not reproductively isolated and frequently exchange propagules with surrounding open coast populations. Indeed, large volumes of kelp from open coast habitats are often washed up inside estuaries after storms [43] indicating that dispersal, at least via this mechanism, is frequent. Tests for first generation migrants confirmed this finding with many putative migrants from estuaries being from the open coast and vice versa. The exception was Port Stephens where kelp populations in estuaries were genetically different from those on the open coast, however, this pattern was driven largely by 1 open coast site (Boat Harbour) and may not be a general pattern. Similarly, in Broken Bay the site that was furthest from the estuarine mouth (Brooklyn) was genetically different from all other sites whether inside or outside the Bay, indicating that in this case, distance or hydrodynamic conditions may limit dispersal from this site.

Despite weak genetic differentiation, spatial patterns of genetic structure appeared to be weakly associated with type of estuary with trends (albeit non-significant) for positive relationships between genetic structure and geographic distance in wave- compared to tide-dominated estuaries. Hydrodynamics within these estuaries and water exchange between the estuaries and nearby open coast sites are vastly different between these different estuarine morphologies and may explain these differences in spatial genetic structuring. In wave dominated estuaries, waves and water motion originating from the open ocean are the dominant structuring influences and this may promote one-way, linear patterns of dispersal. In contrast, tide-dominated estuaries are structured by flushing of tidal currents as water moves back and forth and this may enhance mixing of propagules and prevent the formation of spatial patterns of genetic structure. Although this pattern was only weakly demonstrated here, consideration of the influence of estuarine morphology and associated hydrodynamics on genetic structure may be important for designing MPAs (particularly those in estuarine areas).

Together, these results suggest that the presence of estuaries is not a barrier to dispersal in kelp, either among open coast populations or between estuarine and open coast habitats. Indeed, estuaries may enhance connectivity via provision of additional habitat. Moreover, it is likely that the strength and complex nature of prevailing currents (the East Australian Current) combined with the multiple [3] dispersive stages of this kelp species overrides any influence of local physical setting on patterns of genetic structure. Alternatively, nearshore disturbance regimes characteristic of kelp forests whereby *E. radiata* is cleared from small patches of established forest by storms, grazing or other anthropogenic impacts may result in genetic patchiness because migrants may only be able to colonise bare patches within existing forests or rare areas of unoccupied rocky reef [44], [45]. Regardless, these results suggest that the nature of this coastline with 4 major estuarine systems interspersed along the rocky, open shore need not be treated any differently in terms of connectivity of this habitat forming kelp, than another section of coastline in NSW. However, consideration must be given to obligate estuarine species as well as those that may spend part of their life cycle exclusively in estuaries as such species are likely to exhibit unique patterns of genetic structure and connectivity [46], [14], [47], [48].

These results are particularly pertinent given that the bioregion (Hawkesbury) studied here is the only one in NSW without a marine park. It also represents the most urbanised and developed coastline in Australia and is home to Australia's largest city (Sydney). Protection of marine habitats in this region is currently achieved via numerous small, shallow Aquatic Reserves where marine macrophytes are protected. MPAs may be designated in this region in the future given the need to adequately protect biodiversity along the NSW coastline. Incorporating estuarine areas and open coast into MPA designs and considering linkages among these important habitats, as is currently done in other NSW MPAs, will be key for designing future MPAs in this bioregion. Nearshore and estuarine habitats are inherently complex with a suite of physical and biological factors determining dispersal and connectivity of marine organisms. Teasing apart the relative influence of such factors is important for effective MPA design and to ensure that species and habitats are protected into the future.
